# World Health Organization estimates of the global and regional disease burden of four foodborne chemical toxins, 2010: a data synthesis

**DOI:** 10.12688/f1000research.7340.1

**Published:** 2015-12-03

**Authors:** Herman Gibb, Brecht Devleesschauwer, P. Michael Bolger, Felicia Wu, Janine Ezendam, Julie Cliff, Marco Zeilmaker, Philippe Verger, John Pitt, Janis Baines, Gabriel Adegoke, Reza Afshari, Yan Liu, Bas Bokkers, Henk van Loveren, Marcel Mengelers, Esther Brandon, Arie H. Havelaar, David Bellinger

**Affiliations:** 1 Gibb Epidemiology Consulting LLC, Arlington, VA, USA; 2 Department of Virology, Parasitology and Immunology, Ghent University, Merelbeke, Belgium; 3 Institute of Health and Society (IRSS), Université catholique de Louvain, Brussels, Belgium; 4 Department of Biomedical Sciences, Institute of Tropical Medicine, Antwerp, Belgium; 5 Exponent, Center for Chemical Regulation and Food Safety, Washington, DC, USA; 6 Department of Food Science and Human Nutrition, Michigan State University, East Lansing, MI, USA; 7 Department of Agricultural, Food, and Resource Economics, Michigan State University, East Lansing, MI, USA; 8 National Institute for Public Health and the Environment (RIVM), Bilthoven, Netherlands; 9 Faculdade de Medicina, Universidade Eduardo Mondlane, Maputo, Mozambique; 10 Department of Food Safety and Zoonoses, World Health Organization, Geneva, Switzerland; 11 CSIRO Food and Nutrition Flagship, North Ryde, Australia; 12 Food Data Analysis Section, Food Standards Australia New Zealand, Canberra, Australia; 13 Department of Food Technology, University of Ibadan, Ibadan, Nigeria; 14 Environmental Health Services, British Columbia Centre for Disease Control, Vancouver, BC, Canada; 15 INTERTEK, Oak Brook, IL, USA; 16 Emerging Pathogens Institute and Animal Sciences Department, University of Florida, Gainesville, FL, USA; 17 Institute for Risk Assessment Sciences, Utrecht University, Utrecht, Netherlands; 18 Boston Children’s Hospital, Harvard Medical School, Boston, MA, USA

**Keywords:** public health, epidemiology, foodborne diseases, DALYs, aflatoxin, cassava, cyanide, dioxin, peanut allergen

## Abstract

**Background**

Chemical exposures have been associated with a variety of health effects; however, little is known about the global disease burden from foodborne chemicals. Food can be a major pathway for the general population’s exposure to chemicals, and for some chemicals, it accounts for almost 100% of exposure.

**Methods and Findings**

Groups of foodborne chemicals, both natural and anthropogenic, were evaluated for their ability to contribute to the burden of disease.  The results of the analyses on four chemicals are presented here - cyanide in cassava, peanut allergen, aflatoxin, and dioxin.  Systematic reviews of the literature were conducted to develop age- and sex-specific disease incidence and mortality estimates due to these chemicals.  From these estimates, the numbers of cases, deaths and disability adjusted life years (DALYs) were calculated.  For these four chemicals combined, the total number of illnesses, deaths, and DALYs in 2010 is estimated to be 339,000 (95% uncertainty interval [UI]: 186,000-1,239,000); 20,000 (95% UI: 8,000-52,000); and 1,012,000 (95% UI: 562,000-2,822,000), respectively.  Both cyanide in cassava and aflatoxin are associated with diseases with high case-fatality ratios.  Virtually all human exposure to these four chemicals is through the food supply.

**Conclusion**

Chemicals in the food supply, as evidenced by the results for only four chemicals, can have a significant impact on the global burden of disease. The case-fatality rates for these four chemicals range from low (e.g., peanut allergen) to extremely high (aflatoxin and liver cancer).  The effects associated with these four chemicals are neurologic (cyanide in cassava), cancer (aflatoxin), allergic response (peanut allergen), endocrine (dioxin), and reproductive (dioxin).

## Introduction

Chemicals in food are a worldwide health concern ^1^. Foodborne chemicals, both natural and anthropogenic, have been a source of concern with respect to international trade ^2– 8^, and various articles in the scientific literature have reported the health risks of chemical food contaminants ^9– 11^. The Dutch National Institute for Public Health and the Environment (RIVM) found that chemicals in food contributed as much as infectious agents to the foodborne burden of disease in the Netherlands ^12^.

In September 2006 the World Health Organization (WHO) organized a consultation to develop a strategy to estimate the global burden of foodborne disease ^13^. The first meeting of the WHO Foodborne Disease Burden Epidemiology Reference Group (FERG), convened in September 2007 ^14^, was the first of several meetings ^15– 17^. The FERG includes three hazard-based task forces: Enteric Disease Task Force, Parasitic Disease Task Force, and the Chemical and Toxins Disease Task Force (CTTF). A Country Studies Task Force, a Source Attribution Task Force, and a Computational Task Force were subsequently added to FERG. In the current study, the CTTF reports the estimates of the burden of disease of four chemicals.

## Methods

At its first meeting, the CTTF identified groups of chemicals and toxins that are of highest priority in estimating the burden of foodborne disease. These included:

Elemental contaminants (e.g., lead, mercury, cadmium, manganese, arsenic)
Mycotoxins (e.g., aflatoxins, ochratoxins, fumonisins, trichothocenes)
Food additives (e.g., sulphites, nitrites/nitrates, benzoic acid)
Pesticides/residues (e.g., organophosphates, carbamates, DDT, pyrethrins)
Organic industrial pollutants (e.g., persistent organic pollutants)
Veterinary drugs/residues (e.g., antibiotics, hormones – but not antimicrobial residues)
Seafood toxins (e.g., tetrodotoxin, ciguatera, shellfish toxins, DSPs, PSPs, histamines)
Process contaminants (e.g., acrylamide, PAHs, choropropanol)
Allergens (e.g., peanuts)
Natural toxicants (e.g., cyanide in cassava, aminoglycosides)
Radionuclides and depleted uranium

The hazards were ranked on (1) the severity of potential health effects, (2) the prevalence of exposure, and (3) the availability of data to make burden estimates. After considerable discussion, the final list of chemicals/toxins for which the CTTF believed that burdens could be estimated were aflatoxin, cyanide in cassava, peanut allergen, dioxin and dioxin-like compounds, methylmercury, lead, arsenic, and cadmium. Only the results for aflatoxin, cyanide in cassava, peanut allergen, and dioxin are presented here. The results for the metals will be provided in a subsequent publication.

For each of the four chemicals, a systematic literature review was conducted. It was concluded that burden estimates could be developed for (1) cyanide in cassava and *konzo*; (2) peanut allergy; (3) aflatoxin and hepatocellular carcinoma (HCC); and (4) dioxin and hypothyroidism; and (5) dioxin and decrease in sperm count. The methodology employed for each is described below. Additional information may be found in the Supplementary material.

The metrics used to express burden are those of the WHO ^19^. DALYs are the sum of years lived with disability (YLD) and years of life lost (YLL) ^18^. YLD are estimated from the number of incident cases multiplied by the disability weight (DW) assigned to the disease and the duration of the disease from onset until remission or death ^18^. YLL are estimated from the number of deaths, the distribution of age at death, and life expectancy ^18^. The life expectancy used for the calculations is the projected life expectancy for the year 2050. Estimates of the number of incident cases were produced using United Nations country-level population data for 2010 using the 2012 Revision of World Population Prospects. Uncertainty around input parameters was estimated using Monte Carlo simulations; 10,000 samples from each input parameter were used to calculate 10,000 estimates of cases, deaths or DALYs. The 2.5 ^th^ and 97.5 ^th^ percentile of each set of the 10,000 estimates yielded a 95% uncertainty interval (UI) which is presented around the median ^19^. Detailed information on the input parameters used in the DALY calculations for the different hazards is provided in the Supplementary material.

### Cyanide in cassava

Cassava is an important staple for over 800 million people in approximately 80 countries, mostly in sub-Saharan Africa but also in Asia, the Pacific, and South America ^20^. Cassava tubers contain a varying quantity of cyanogenic glucosides which protect the root against attack by animals and insects. Appropriate processing before consumption can reduce cyanogenic glucoside content of cassava. When high cyanogenic cassava is not processed correctly, high dietary cyanide exposure occurs. This often happens during times of famine and war. Cyanide in cassava is associated with acute cyanide poisoning and several diseases including *konzo* ^21^. Worldwide reports exist of acute poisoning from cyanide in cassava ^21^ exist, but the data are inadequate to make burden estimates. The data are sufficient, however, to make burden estimates of *konzo*. *Konzo* is an irreversible spastic paraparesis of sudden onset, associated with the consumption of bitter cassava ^22, 23^ and a low protein intake ^24^. It is a disease of extreme poverty. *Konzo* mostly occurs in epidemics, but sporadic cases are also reported. The case definition includes the following criteria: (1) a visible symmetrically spastic abnormality of gait while walking and/or running; (2) a history of abrupt onset (less than one week), followed by a non-progressive course in a formerly healthy person; (3) bilaterally exaggerated knee and/or ankle jerks without signs of disease in the spine ^24, 25^.

Because *konzo* mostly affects remote rural areas where health infrastructure is poor or non-existent, many cases remain undiagnosed or unreported, so the true burden of disease remains unknown. No cases have been reported from urban areas. A total of 2376 *konzo* cases have been reported in 5 countries in Africa (Cameroon, Central African Republic, Democratic Republic of Congo [DRC], Mozambique, and United Republic of Tanzania) ^21^, corresponding to 149 cases per year for 122 million people. Dividing the average annual number of cases for each country by the corresponding country population produces an observed incidence ranging from 0.043 to 0.179 per 100,000. The degree of underestimation is difficult to determine as *konzo* occurs in rural areas, often under conditions of war, and the disease is not notifiable. The only reported calculation of underestimation was that of Tylleskar ^25^ in the DRC in 1994, when he estimated that at least twice as many cases may have occurred as those reported. The underestimation in the DRC is likely to be much greater more recently, due to war and displacement. It was therefore decided to account for the uncertainty in the underreporting by applying an expansion factor ranging uniformly from 1 to 10 to the observed cases. The mean annual incidence rate was therefore estimated as 0.9/100,000 (0.04 to 1.8/100,000). Our estimate of the burden of *konzo* is restricted to the 5 African countries described above and Angola. The decision to include Angola is based on a report to the World Congress on Neurology suggesting that cases have occurred in that country ^26^. Although cassava consumption occurs in tropical areas throughout the world, the term *konzo* has only been used to describe cases in Africa. The incidence of *konzo* in other countries in Africa and other parts of the world is assumed to be zero.

We assumed the age of onset and gender distribution of these cases to be that observed by Tylleskar ^25^. The *konzo* case-fatality ratio is approximately 21% based on four studies ^25, 27– 29^. The age and gender distribution of fatal cases was assumed to be that of Tshala-Katumbay ^27^.

The onset of paraparesis in *konzo* is abrupt, usually within minutes or hours, with occasional progression during the first days of the illness. After that time, the paraparesis is non-progressive and permanent. As a result, duration is defined as lifelong for non-fatal cases. For fatal cases, it was assumed that death occurred one to seven years after onset, with a most likely value of three years after onset, following Banea *et al.* ^28^ and Tylleskar *et al.* ^30^.

There is no DW specifically for *konzo*. The WHO defined three severity levels for *konzo*: (1) Mild = able to walk without support; (2) Moderate = uses one or two sticks or crutches to walk; and (3) Severe = not able to walk ^24^. The Global Burden of Disease (GBD) 2010 DWs for mild, moderate, and severe motor impairment are 0.012, 0.076, and 0.377, respectively ^31^. The distribution of *konzo* severity among 753 patients from nine different studies were mild (63%), moderate (27%) and severe (10%) ^27, 28, 30, 32– 37^. This distribution and the disability weights described above were used to assign a disability weight of 0.065 to *konzo*.

### Peanut allergen

Prevalence data on peanut allergy were used to make estimates of incidence since allergy occurs early in life (< 5years) and is believed to be lifelong ^38– 42^. All peanut allergy cases are assumed to be the result of eating peanuts or peanut products. In western countries, the prevalence of clinical peanut allergy in children is 0 to 1.8% of the population ^38^, corresponding to incidence rates of 0 to 22.6 per 100,000. Limited data exist on the mortality rate of peanut-induced anaphylaxis, but the majority of studies found similar rates, ranging from 0 to 0.006 deaths per 100,000 person-years ^38^. Incidence was estimated only for the WHO A level (high income) subregions; too few data exist to make estimates for other subregions ^38^. Several studies have reported that 63–66% of cases are male ^38^, but given the uncertainty in this number, the gender distribution was assumed to be equal for the burden of disease calculations. No DW exists for peanut allergy. Mullins *et al.* ^39^ reported that 52% of cases referred to a specialist allergy medical practice in Australia suffered from mild symptoms (skin and subcutaneous tissue involvement only), 42% from moderate symptoms (features suggestive of respiratory, cardiovascular or gastrointestinal involvement), and 6% from severe symptoms (cyanosis, hypotension, confusion, collapse, loss of consciousness, incontinence). We propose the DW for peanut allergy be a weighted average accounting for this severity distribution. GBD 2010 DWs ^31^ for the health states defined in the category “Asthma: controlled” (DW=0.009) are considered applicable for mild and moderate cases (94%), and “Generic uncomplicated disease: anxiety about the diagnosis” (DW=0.054) for severe cases (6%), because anxiety is known to impact quality of life in food allergic patients ^43^, leading to a severity-weighted DW of 0.012 for clinically relevant peanut allergy. Unlike other childhood allergies such as cow’s milk and egg allergy, peanut allergy rarely resolves ^44, 45^.

### Aflatoxin

Aflatoxins are secondary metabolites of the fungi *Aspergillus flavus* and *A. parasiticus*, and less frequently other *Aspergillus* species such as *A. nomius* ^46^. These species can be found in maize, peanuts (groundnuts), oilseeds, and tree nuts in tropical and subtropical regions ^46^. It is believed that all aflatoxin exposure results from food consumption. We assumed a multiplicative model for the effects of aflatoxin exposure and hepatitis B virus (HBV) infection and estimated excess risk due to aflatoxin exposure as described by Liu and Wu ^46^. To account for differences in background rates between the study population from which the cancer potency factor was derived ^47^ and global populations, we estimated population attributable fractions (PAFs) by country, and applied them to HCC incidence and mortality based on ^48, 49^. A Bayesian log-normal random effects model ^50^ was used to extrapolate available PAFs to countries without data. Age-specific incidence estimates were derived from a study in China comparing age-specific incidence of HCC in Qidong, a city in China with high aflatoxin exposure, and Beijing, a city with low aflatoxin exposure ^51^. The YLD and YLL envelopes for HCC that are available from WHO were multiplied by the proportion of the burden due to aflatoxin. Thus no DW was directly involved in the calculation.

### Dioxin

Dioxins are mainly byproducts of industrial processes, but can also result from natural phenomena such as volcanic eruptions and forest fires. More than 90% of human exposure to dioxins is through the food supply. The foods most often associated with dioxin contamination are meat, dairy products, fish, and shellfish ^52^. Due to the bioaccumulation and lipophilic characteristics of dioxins, daily dietary exposure leads to accumulation of these compounds in human body fat. In adults this accumulation is thought to reach a constant level (i.e., a steady state). Consequently, the dioxin body burden, rather than the daily exposure, is taken as the dose metric for chronic toxicity risk and the assessment of dioxins ^53– 58^. In this context the dioxin concentration in breast milk fat directly reflects the concentration in body fat ^58– 61^.

Many national authorities have programs in place to monitor dioxin in the food supply and breast milk ^61– 63^. Dioxin-induced prenatal and postnatal hypothyroidism and prenatally induced reduced sperm production have been found to be the most sensitive non-cancer toxic endpoints for dioxins. Estimates for dioxin-induced prenatal and postnatal hypothyroidism and reduced fertility due to disturbed sperm formation were based on an exposure assessment, toxicity assessment, and the comparison of both assessments ^64, 65^. The exposure assessment is based on breast milk concentrations of dioxin from 50 countries ^63^. The toxicity assessment utilizes the benchmark dose (BMD) approach ^66– 68^ in which the dose response of postnatal total thyroxine (TT; decrease of TT4 in adult blood), prenatal thyroid stimulating hormone (TSH; increase in TSH in neonatal blood), and sperm production (reduced concentration of sperm cells) is analyzed. The toxicity and exposure assessments are compared to derive the transgression of a dioxin induced decrease in TT4, decrease in sperm cell count and increase in TSH across a physiological threshold indicating a disease status (i.e., incidence of hypothyroidism or impaired fertility). Additional details of these assessments may be found in Zeilmaker *et al.* ^69^. The BMD analysis was performed on studies which served as the starting point for the derivation of a tolerable weekly intake (TWI) ^54– 57^ or reference dose for dioxin (RfD) ^58^.

In a study of a mother-child cohort, Baccarelli *et al.* determined the relationship between maternal plasma dioxin concentration and TSH level ^70^. A BMD analysis of these data resulted in a population distribution of the maternal body burden of dioxin corresponding to an increased TSH level of 5 µU/mL in offspring, a level not to be exceeded in 3% of newborns in iodine-replete populations ^71^.

Following administration of an acute oral dose to pregnant Long Evans rats on day 15 of gestation, Gray *et al.* measured the reduction in cauda epididymis sperm count in male offspring ^72^. The resulting dose response data were used to calculate a BMD lower confidence limit (BMDL) and upper confidence limit (BMDU) dioxin body burden for various levels of reduction in sperm count. A WHO reference cut-off value for impaired fertility of 20 × 10 ^6^ sperm cells/mL was used to link toxicity (sperm count reduction) to a disease status (impaired fertility) (i.e., the calculation of the probability of a male being born with dioxin-impaired fertility) ^73^.

A BMD analysis of a National Toxicology Program (NTP) two year feeding study in rats was used to make estimates of dioxin-induced thyroid toxicity. The NTP study administered 2,3,7,8-tetrachlorodibenzo-p-dioxin (TCDD) ^74^ and 2,3,4,7,8-pentachlorodibenzofuran ^75^ for periods of 14, 31, and 53 weeks. The concentrations were converted to toxic equivalent quotients ^76^ to enable a combined analysis of both congeners. BMDL and BMDU body burdens for reduction in TT4 were calculated for each of the exposure periods. A distribution of TT4 in human blood has been reported by Aoki *et al.* ^71^. The 5 ^th^ percentile of this distribution (65 nmol/L) was used as the cut-off for overt clinical hypothyroidism in adults.

The results of the BMD analyses and the breast milk concentrations for 50 countries were compared, taking account of possible differences between experimental animals and humans and among individual humans ^64, 65^. This comparison provided country-specific estimates of the incidence of dioxin induced prenatal and postnatal hypothyroidism and impaired fertility. The estimates were extrapolated to other countries for which no breast milk concentrations were available by means of Bayesian random effects modeling ^50^.

## Results

Raw data for Gibb *et al.* 2015, ‘World Health Organization estimates of the global and regional disease burden of four foodborne chemical toxins, 2010’ A detailed description of the data can be found in the text file provided (‘Raw data legends’). Click here for additional data file. Copyright: © 2015 Gibb H et al. 2015 ${data-license-text}

The analyses presented here show that four selected chemicals already have a substantial impact on the foodborne burden of disease, particularly in low- and middle-income countries. Just these four agents are estimated to be associated with 339,000 illnesses (95% UI: 186,000–1,239,000); 20,000 deaths (95% UI: 8,000–52,000); and 1,012,000 DALYs (95% UI: 562,000–2,822,000), respectively, in the year 2010. These should be considered the “tip of the iceberg” in terms of foodborne chemicals and their impact on the global burden of disease. For peanut allergens, we were unable to estimate a burden for low- and middle-income countries due to data gaps. We also had to use an approximate disability weight, as there are data only on quality of life of patients with food allergy ^38^ and no specific data are available for peanut allergy.

The estimated number of incident cases, deaths, and DALYs of each of the diseases associated with chemicals is given in Table 1. The chemical associated with the most number of illnesses is dioxin; however, no deaths have been reported from the presence of dioxin in the food supply. The chemical associated with the greatest number of DALYs is aflatoxin. The DALY estimates for aflatoxin and dioxin have the least uncertainty; more uncertainty is associated with the DALY estimates for peanut allergen and cyanide in cassava. The annual incidence, mortality, and DALY rate of each chemical-associated disease per 100,000 population for each of the WHO regions is reported in Table 2. Peanut allergy is not reported in Table 2 because burden was estimated only for Americas Region A (AMR A) - United States, Canada, and Cuba); Europe A (EUR A) - primarily countries in western Europe; and Western Pacific Region A (WPR A) - Australia, Brunei Darussalam, Japan, and New Zealand. Burden estimates for cyanide in cassava are provided only for the African region (AFR) and assumed to be zero for other regions.

**Table 1.** Median number of foodborne illnesses, deaths, and DALYs, with 95% UIs, 2010.

CHEMICAL	FOODBORNE ILLNESSES (95% UI)	FOODBORNE DEATHS (95% UI)	FOODBORNE DALYS (95% UI)
Aflatoxin	21,757 (8,967–56,776)	19,455 (7,954–51,324)	636,869 (267,142–1,617,081)
Cyanide in cassava	1,066 (105–3,016)	227 (22–669)	18,203 (1,769–53,170)
Dioxin	193,447 (155,963–1,085,675	0 (0–0)	240,056 (192,608–1,399,562)
Peanut allergens*	107,167 (6,262–210,093)	28 (2–56)	99,717 (5,827–195,489)
**TOTAL**	338,611 (185,705–1,238,725	19,736 (8,210–51,700)	1,012,362 (562,087–2,822,481)

*Only the burden for AMR A, EUR A, and WPR A was assessed.

**Table 2.** Median rate per 100,000 foodborne illnesses, deaths, and DALYs by WHO region, with 95% UIs.

REGION		CHEMICAL			
		Aflatoxin	Cyanide in Cassava	Dioxin	Total
AFRO	FB Illnesses (95% CI)	0.4 (0.1–1)	0.1 (0.01–0.4)	0.2 (0.07–7)	0.7 (0.3–8)
	FB Deaths (95% CI)	0.4 (0.1–1)	0.03 (0.003–0.08)	0 (0–0)	0.4 (0.1–1)
	FB DALYs (95% CI)	15 (5–40)	2 (0.2–6)	0.2 (0.07–8)	18 (7–49)
AMRO	FB Illnesses (95% CI)	0.08 (0.02–0.6)	0 (0–0)	0.2 (0.05–6)	0.2 (0.1–7)
	FB Deaths (95% CI)	0.08 (0.02–0.6)	0 (0–0)	0 (0–0)	0.08 (0.02–0.6)
	FB DALYs (95% CI)	2 (0.4–15)	0 (0–0)	0.2 (0.07–9)	2 (0.6–24)
EMRO	FB Illnesses (95% CI)	0.2 (0.04–0.5)	0 (0–0)	2 (1–35)	2 (1–35)
	FB Deaths (95% CI)	0.1 (0.04–0.4)	0 (0–0)	0 (0–0)	0.1 (0.04–0.4)
	FB DALYs (95% CI)	4 (1–13)	0 (0–0)	2 (2–43)	7 (3–51)
EURO	FB Illnesses (95% CI)	0.02 (0.01–0.03)	0 (0–0)	1 (0.7–13)	1 (0.7–13)
	FB Deaths (95% CI)	0.02 (0.01–0.03)	0 (0–0)	0 (0–0)	0.02 (0.01–0.03)
	FB DALYs (95% CI)	0.5 (0.3–0.8)	0 (0–0)	1 (0.9–19)	2 (1–19)
SEARO	FB Illnesses (95% CI)	0.2 (0.08–0.6)	0 (0–0)	9 (8–32)	10 (8–32)
	FB Deaths (95% CI)	0.2 (0.08–0.5)	0 (0–0)	0 (0–0)	0.2 (0.07–0.5)
	FB DALYs (95% CI)	7 (2–17)	0 (0–0)	12 (10–41)	19 (13–54)
WPRO	FB Illnesses (95% CI)	0.6 (0.1–2)	0 (0–0)	0.05 (0.005–4)	0.8 (0.1–5)
	FB Deaths (95% CI)	0.5 (0.09–2)	0 (0–0)	0 (0–0)	0.5 (0.09–2)
	FB DALYs (95% CI)	16 (3–63)	0 (0–0)	0.07 (0.007–6)	16 (3–65)
**GLOBAL**	FB Illnesses (95% CI)	0.3 (0.1–0.8)	0.02 (0.002–0.04)	3 (2–16)	3 (3–17)
	FB Deaths (95% CI)	0.3 (0.1–0.7)	0.003 (0–0.01)	0 (0–0)	0.3 (0.1–0.8)
	FB DALYs (95% CI)	9 (4–24)	0.3 (0.03–0.8)	3 (3–20)	13 (7–39)

**Figure 1.**
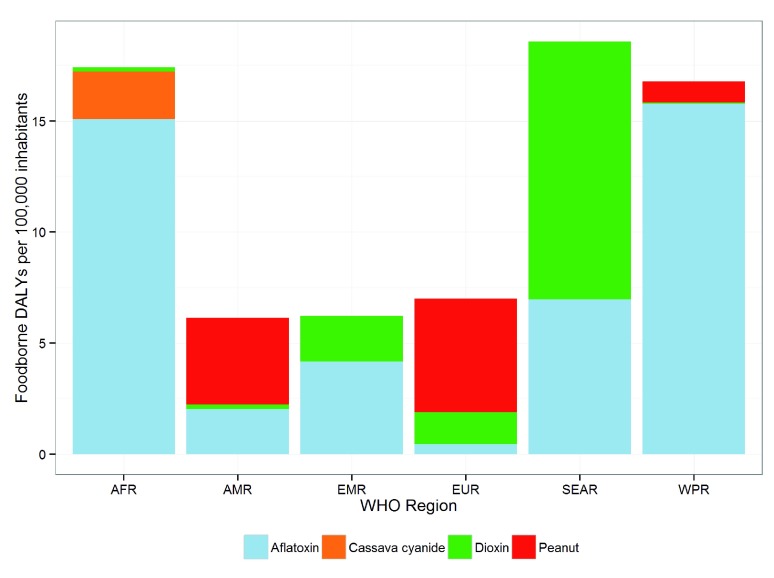
The relative contribution to the DALY incidence by each of four chemicals for each of the WHO regions.

**Figure 2.**
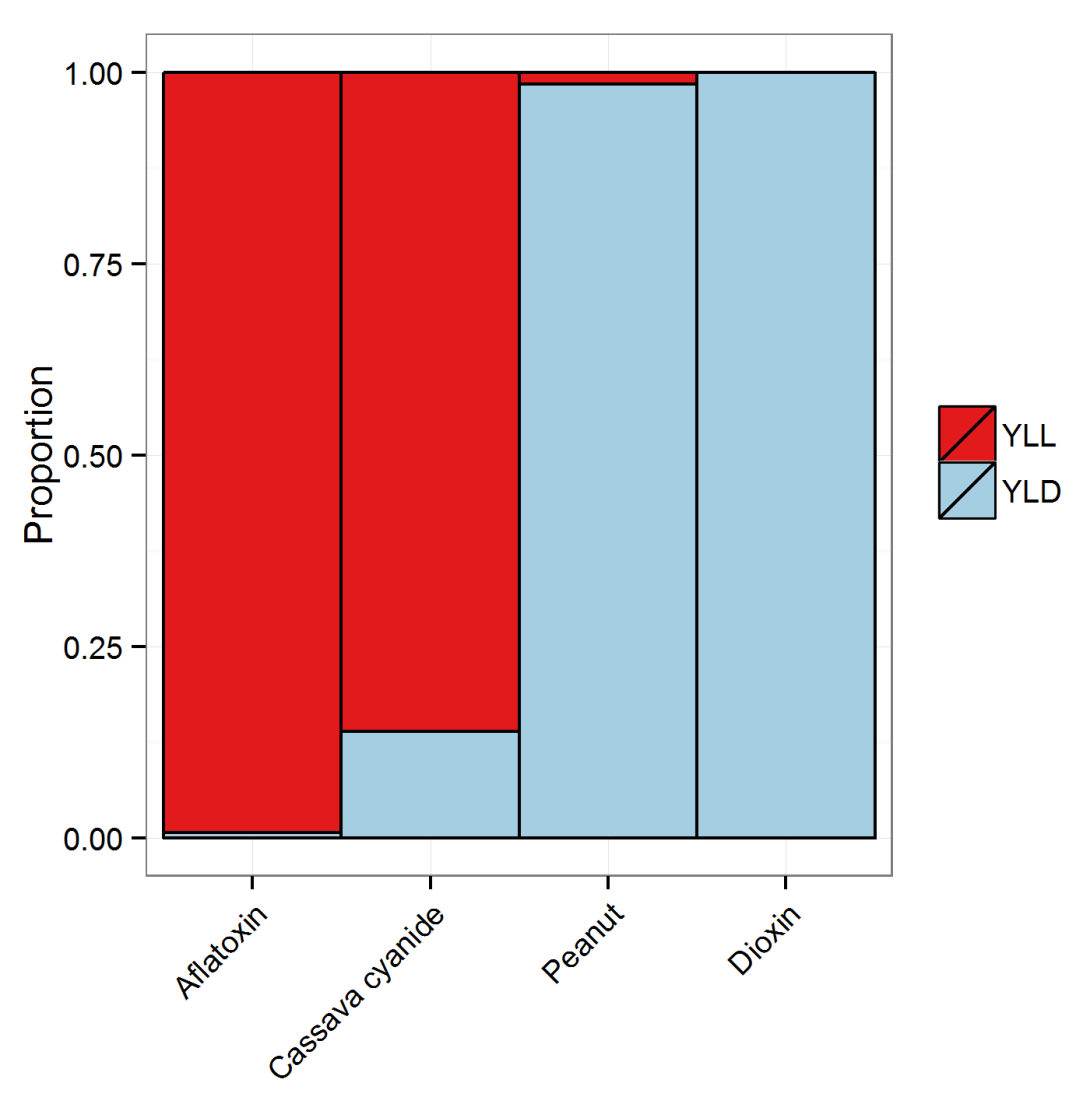
The relative contributions from YLLs and YLDs for each of four chemicals.

**Figure 3.**
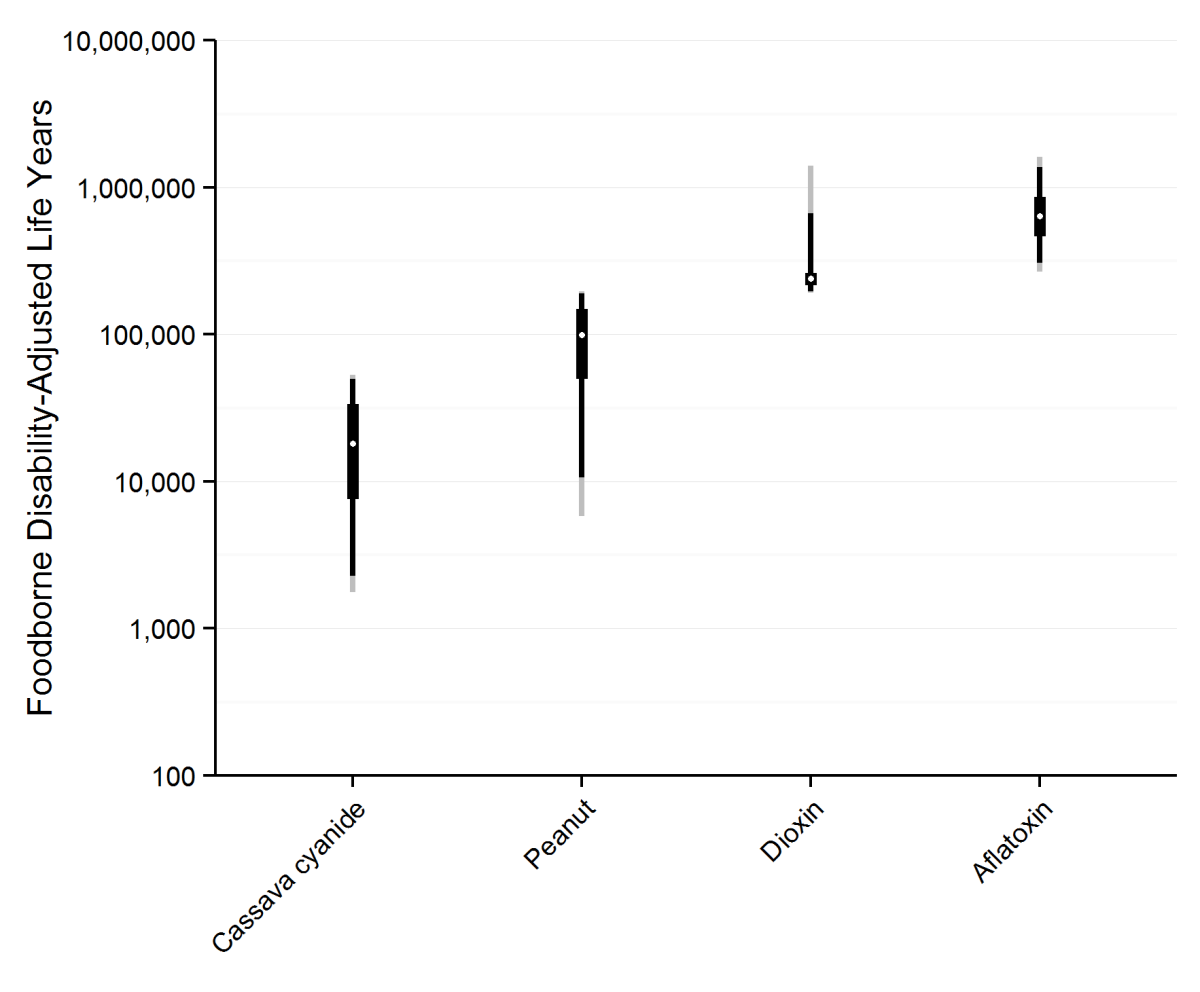
DALY for each of four chemicals from contaminated food ranked from lowest to highest with 95% UI (The dot in the middle of each box represents the median, the box the 50% UI, the dark bar the 95% UI, and the light bar the 95% UI).

Figure 1 provides the DALYs per 100,000 inhabitants by global region. The regions with the highest burden per 100,000 inhabitants are the Southeast Asia Region (SEAR), Western Pacific Region (WPR), and the African Region (AFR). The AMR, Eastern Mediterranean Region (EMR), and EUR have the lowest DALYs per 100,000. Aflatoxin is the largest contributor to the burden in AFR and WPR. Dioxin makes the largest contribution in SEAR. Figure 2 contrasts the proportion of DALYs due to YLL and YLD for each of the four chemicals. Virtually all of the DALYs for aflatoxin and most of the DALYs for cyanide in cassava are due to YLL, whereas most of the DALYs for peanut allergen and all of the DALYs for dioxin are due to YLD. Figure 3 shows the uncertainty around the DALY estimates for each of the four chemicals. The chemical with the least uncertainty and the most number of DALYs is aflatoxin.

## Discussion

The assessment of burden of disease from chemicals in the food is a challenge on several levels. There are thousands of chemicals in production and many naturally occurring toxins. How many of these chemicals and toxins make it into the food supply is unknown. The health effects of chemicals may not be observed for years following exposure (e.g., aflatoxin and liver cancer, lead and cardiovascular disease). Longitudinal studies of these effects are expensive and time-consuming. Sufficient information is available, however, to make estimates of the burden for arsenic, cadmium, methyl mercury, and lead and possibly for other chemicals and toxins (e.g., fish toxins, aristolochic acid). Other chemicals (e.g., persistent organic pollutants) may not require elaborate epidemiological studies because the burden can be derived from biomonitoring data in combination with relevant toxicity data. Estimates of the burden for these chemicals will provide a much more comprehensive understanding of the impact that chemicals in the food supply have on the burden of disease.

As the relevant disease endpoints due to foodborne chemicals may arise from different causes, various approaches are possible for estimating incidence and mortality. A “top-down” approach uses an existing estimate of morbidity or mortality of the disease endpoint by all causes (“envelope”) as a starting point. A population attributable fraction is then calculated for the hazard under consideration, and applied to the envelope to estimate the hazard-specific incidence. This method, which is the standard in global burden of disease estimations, was used for aflatoxin. A “bottom-up” or dose response approach uses dose-response and exposure information. The approach begins with selection of the appropriate dose response relationship between the chemical and the particular disease. This dose response relationship is then combined with the distribution of exposure within a population to derive an estimate of the incidence of the disease that is attributable to the exposure. A probabilistic version of this method, which is applied in chemical risk assessment, was used for dioxin ^64, 65^. The two approaches would result in the same results if perfect data were available, and if it can be assumed that the risk of exposure to a chemical is additive to the background risk from other causes. In reality, the available data for both approaches are limited and there is insufficient information to decide conclusively whether risks are additive, multiplicative or otherwise. This may result in considerable discrepancies between results from these methods. In this study, we chose a “top-down” approach for aflatoxin because the cancer potency factor derived by the Joint FAO/WHO Expert Committee on Food Additives (JECFA) ^47^ was based on a multiplicative model, and there is evidence for a high background rate in the study population underlying this estimate and the global population (see Supplementary material). Using the population attributable fraction approach, we estimated there were approximately 22,000 (95% UI 9,000–57,000) cases of aflatoxin-related HCC in 2010. A dose response approach ^46^ estimated that annually, 25,200–155,000 cases of HCC may be attributable to aflatoxin exposure. Even though the uncertainty intervals overlap, there is significant difference between these two approaches. There is evidence for a high background rate in the study population underlying this estimate and the global population (see Supplementary material), which may result in overestimation of mortality by the dose response approach. On the other hand, the global liver cancer envelope may be underestimated, particularly in Africa ^77, 78^, leading to underestimation of the aflatoxin attributable incidence.

It is hoped that the presentation here will raise awareness among countries planning their own foodborne burden of disease assessments to consider natural and anthropogenic chemicals. It is also hoped that this publication will lead to the development of chemical specific biomonitoring data to assess exposure and of epidemiologic data on other diseases associated with chemicals in food.

## Data availability

The data referenced by this article are under copyright with the following copyright statement: Copyright: © 2015 Gibb H et al.

*F1000Research*: Dataset 1. Raw data for Gibb *et al.* 2015, ‘World Health Organization estimates of the global and regional disease burden of four foodborne chemical toxins, 2010’, 10.5256/f1000research.7340.d107254 ^79^

## Supplementary material

Incidence, clinical outcomes, duration, disability weights, mortality, age and sex distribution of 4 chemicals/toxins transmitted through food.
