# Supplementary material for: World Health Organization estimates of the global and regional disease burden of four foodborne chemical toxins, 2010: a data synthesis
Source: F1000Res. 2015 Dec 3;4:1393. [Version 1] doi: 10.12688/f1000research.7340.1 (PMC4755404; doi:10.12688/f1000research.7340.1)
Supplement: Supplementary file 2 [file f1000research-4-7910-s0001.tgz › d964f6fb-c237-45d9-bcfe-5eb674080f22.docx]

World Health Organization estimates of the global and regional disease burden of four foodborne chemicals and toxins, 2010

Supplementary material

# Table of Contents

[Aflatoxin 2](#_Toc416450263)

[Cyanide cassava 4](#_Toc416450264)

[Dioxin 6](#_Toc416450265)

[Peanut allergens 8](#_Toc416450266)

# Aflatoxin

| **Incidence** | A population attributable fraction (PAF) approach was used to estimate the incidence of aflatoxin-related hepatocellular carcinoma (HCC). We assumed a multiplicative model for the effects of aflatoxin exposure and hepatitis B virus (HBV) infection. The excess risk due to aflatoxin exposure is estimated as HCC_a-_ = b * a for HBV-negative individuals and HCC_a+_ = b * h * a for HBV-positive individuals, where a = exposure to aflatoxin (ng/(kg bw * day)), b = aflatoxin cancer potency factor in HBV- individuals ((ng/(kg bw * day)^-1^) and h = relative risk for aflatoxin exposure in HBV+ individuals compared to HBV- individuals. We used potency factors as derived by JECFA [1]: b = 0.01 [0.002-0.03](ng/(kg bw * day)^-1^ and b * h = 0.30 [0.005-0.50](ng/(kg bw * day)^-1^. Uncertainty in the potency factors was modeled as a Gamma distribution with the most likely value as the mean and the range representing an approximate 95% confidence interval.  To account for differences in background rates between different populations, we estimated PAFs by country, and applied them to HCC incidence based on [2]. We assumed PAFs for incidence and deaths were equal and calculated PAFs based on published studies on HCC mortality. For the study population that was the basis of the JECFA potency estimates in Guangxi, China [3], the PAF was estimated as PAF_a_ = ((1-p) * HCC_a-_ + p * HCC_a+_)/HCC, where p = prevalence of HBV infection and HCC = total incidence of HCC by all causes. We used the HCC death rate for the Guangxi cohort standardized to the global population (121.5 per 100,000) and calculated average exposure (607 ng/(kg bw * day) based on [4, Table I]. HBV prevalence was 23% based on [3], resulting in PAF_a_ = 0.383. Background death rate of HCC by all causes in the Guangxi population was calculated as HCC_0,s_ = (HCC_s_ – HCC_a,s_) / (1-p_s_ + h * p_s_), with the subscript s referring to the study population; resulting in HCC_0,s_ = 9.77 per 100,000.  To calculate attributable incidence in all countries, we estimated relative risks due to aflatoxin exposure as RR_a,c_ = 1 + b * a_c_ / HCC_0,s_, and PAFs per country as PAF_a,c_ = (RR_a,c_ – 1) / RR_a,c_, with the subscript c indicating country. Attributable incidence was then calculated as HCC_a,c_ = HCC_c_ * PAF_a,c_. Aflatoxin exposure by country was based on [5], with uncertainty represented by a uniform distribution over the reported range. A Bayesian log-normal random effects model [6, 7] was used to extrapolate available PAFs to countries without data. |
| --- | --- |
| **Clinical outcomes** | Hepatocellular carcinoma. |
| **Duration** | Not applicable; population attributable fractions as described above were directly applied to WHO YLD estimates [8]. |
| **Disability Weight** | Not applicable; population attributable fractions as described above were directly applied to WHO YLD estimates [8]. |
| **Mortality** | Population attributable fractions as described above were directly applied to WHO mortality estimates [8]. |
| **Age distribution** | We compared age distributions of HCC in the populations of Beijing and Qidong from [9], and hypothesized that the main difference in HCC risk factors between these two cities is aflatoxin exposure, since every other risk factor is the same, and they are both predominantly Han (i.e., same ethnicity). Hence, the difference in age distributions was presumed to be the contribution of aflatoxin. This resulted in an average age at onset of 49. |
| **Sex distribution** | In absence of information on the sex distribution of aflatoxin-induced hepatocellular carcinoma, a 50:50 age distribution was assumed. |

**References**

1. International Programme on Chemical Safety. Safety eval­uation of certain food additives and contaminants. Aflatoxin. WHO Food Additives Series 40. World Health Organization, Geneva, 1998.
2. GLOBOCAN (2012) GLOBOCAN 2012: Estimated cancer incidence, mortality and prevalence worldwide in 2012. <http://globocan.iarc.fr/Pages/fact_sheets_cancer.aspx>, accessed on 10/04/2015.
3. Yeh FS, Yu MC, Mo CC, Luo S, Tong MJ, Henderson BE (1989). Hepatitis B virus, aflatoxins, and hepatocellular carcinoma in southern Guangxi, China. Cancer Res 49(9):2506–2509.
4. Wu-Williams AH, Zeise L, Thomas D (1992) Risk assessment for aflatoxin B1: A modeling approach. Risk Anal, 12: 559-567.
5. Liu Y, Wu F (2010) Global burden of aflatoxin-induced hepatocellular carcinoma: a risk assessment. Environmental Health Perspectives 118(6): 818-824.
6. Devleesschauwer B, Haagsma JA, Angulo FJ, Bellinger D, Cole D, Döpfer D, et al. (2015) Methodological framework for World Health Organization estimates of the global burden of foodborne disease. In preparation.
7. McDonald SA, Devleesschauwer B, Speybroeck N, Hens N, Praet N, Torgerson PR, Havelaar AH, Wu F, Tremblay M , Amene EW, Döpfer D. Data-driven methods for imputing national-level incidence rates in global burden of disease studies (2015) Bull World Health Org 93:228-236.
8. World Health Organization. Global Health Estimates by Cause, Age, Sex and Country, 2000-2012. Geneva, WHO, 2015. Available from: <http://www.who.int/entity/healthinfo/global_burden_disease/>, accessed on 10/04/2015.
9. Kensler TW, Qian G-S, Chen J-G, Groopman JD (2003) Translational strategies for cancer prevention in liver. Nature Reviews Cancer, 3: 321-329.

# Cyanide cassava

| **Incidence** | A total of 2376 *konzo* cases have been reported in 5 countries (Cameroon, Central African Republic, Democratic Republic of Congo, Mozambique, and United Republic of Tanzania), corresponding to 149 cases per year for 122 million people [1]. Based on these cases and dividing the average annual number of case for each country by the corresponding country population is giving an observed incidence of 0.043 to 0.179 per 100,000.  The degree of underestimation is difficult to estimate, as konzo occurs in remote rural areas, often under conditions of war, and the disease is not notifiable. The only previous calculation of underestimation was that of Tylleskar [2] in the DRC in 1994, when he estimated that there may have been at least twice as many cases as those reported. The underestimation in the DRC is now likely to be much greater, due to war and displacement. It was decided to account for the uncertainty in the underreporting by applying an expansion factor ranging from 1 to 10 to the observed cases. Therefore, the annual total of new cases would range from 149 to 1490 in the 5 countries and the mean annual incidence rate would be 0.9/100,000 (0.04 to 1.8/100,000).  We restricted our estimates of konzo disease to the 5 African countries in which the disease has been reported and Angola, based on a report to the World Congress on Neurology suggesting that cases have occurred in that country [3]. The incidence of konzo disease was assumed to be null in other countries around the world. |
| --- | --- |
| **Clinical outcomes** | Konzo disease is a paraparesis occurring in populations exposed to cyanogenic glycoside in a context of bitter cassava consumption associated with a low intake of protein-rich food. |
| **Duration** | The onset of paraparesis is abrupt, usually within minutes or hours, with occasional progression during the first days of the illness. After that time, the paraparesis is non-progressive and permanent. As a result, duration was defined as lifelong for non-fatal cases. For fatal cases, it was assumed that death occurred one to seven years after onset, with an average of three years after onset, following [3,4]. |
| **Disability Weight** | No specific DW exists for konzo paraparesis. WHO [5] defined three severity levels for konzo:  1. Mild = Able to walk without support  2. Moderate = uses one or two sticks or crutches to walk  3. Severe = not being able to walk  These three severity levels can be matched with the GBD 2010 health states *Motor impairment: mild* (DW=0.012), *Motor impairment: moderate* (DW=0.076), and *Motor impairment: severe* (DW=0.377) [6].  Information on the distribution of konzo severity levels is available from 9 studies [1]. Out of a total of 753 cases, 476 (63%) were mild, 203 (27%) were moderate and 74 (10%) were severe.  The resulting weighted DW equaled 0.065. |
| **Mortality** | Information on case fatality was provided in 4 studies [4,7,8,9]. Out of a total of 340 cases, 73 deaths were observed, yielding an average case fatality ratio of 21%. |
| **Age distribution** | The age and sex distribution observed by [2] was generalized to the whole konzo affected population. The age and sex distribution for fatal cases was adapted from [7]. |
| **Sex distribution** | The age and sex distribution observed by [2] was generalized to the whole konzo affected population. The age and sex distribution for fatal cases was adapted from [7]. |

**References**

1. Cliff J (2011) Incidence and prevalence estimates of cassava cyanide-induced diseases. WHO/FERG report, pp. 75.
2. Tylleskar T (1994) The causation of konzo. Studies on a paralytic disease in Africa. Department of Pediatrics, International Child Health Unit, Uppsala University and Department of Epidemiology and Public Health, Umea University. Uppsala, Uppsala University, pp. 108.
3. Bettencourt Mateus MS, Paquisse MM, Zangulo A, Resende I. 2011. Tropical spastic paraparesis; a major neurologic problem in Caungulia Angola related to of cassava: first report from Angola. XXth World College of Neurology. Marrakesh.
4. Tylleskar T et al. (1991) Epidemiological evidence from Zaire for a dietary etiology of konzo, an upper motor neuron disease. Bull World Health Organ. 69(5): 581-589.
5. Banea M et al. (1992) [High prevalence of konzo associated with a food shortage crisis in the Bandundu region of Zaire]. Ann Soc Belg Med Trop. 72(4): 295-309.
6. WHO (1996) Konzo, a distinct type of upper motor neuron disease. Wkly Epidemiol Rec. 71: 225-232.
7. Salomon JA, Vos T, Hogan DR, Gagnon M, Naghavi M, Mokdad A, et al. (2012) Common values in assessing health outcomes from disease and injury: disability weights measurement study for the Global Burden of Disease Study 2010. Lancet 380(9859): 2129-2143.
8. Tshala-Katumbay D et al. (2001) Neuroepidemiology of konzo - a spastic paraparesis of acute onset in a new area of the Democratic Republic of Congo. Afr J Neurol Sci. 20: 8-12.
9. Ministry of Health, Mozambique (1984) Mantakassa: an epidemic of spastic paraparesis associated with chronic cyanide intoxication in a cassava staple area of Mozambique. 1. Epidemiology and clinical and laboratory findings in patients. Bull World Health Organ. 62(3): 477-484.

# Dioxin

| **Incidence** | Incidence rates were generated for 50 countries and specified as lower and upper bounds (hypothyroidism-postnatal & male infertility) or point estimates (hypothyroidism-prenatal). Incidence rates for the remaining 144 countries were imputed using a Bayesian log-normal random effects model [1]. |
| --- | --- |
| **Clinical outcomes** | Hypothyroidy due to prenatal exposure; hypothyroidy due to postnatal exposure; male infertility due to prenatal exposure |
| **Duration** | Hypothyroidy was assumed to be lifelong; the impact of male infertility was assumed to be present in the 20-44 age group, in accordance with [2]. |
| **Disability Weights** | 1. **Hypothyroidism due to prenatal exposure**   0.019; corresponding to GBD 2013 health state *Hypothyroidy* [3]. (Note that no corresponding DW was available in GBD2010 or WHO GHE)   1. **Hypothyroidism due to postnatal exposure**   0.019; corresponding to GBD 2013 health state *Hypothyroidy* [3]. (Note that no corresponding DW was available in GBD2010 or WHO GHE)   1. **Male infertility**   0.056; corresponding to WHO GHE health state *Infertility: primary* [4]. (Note that this is higher than the corresponding GBD 2010 health state) |
| **Mortality** | No mortality was assumed. |
| **Age distribution** | 1. **Hypothyroidism due to prenatal exposure**   Onset = birth   1. **Hypothyroidism due to postnatal exposure**   Onset = 20   1. **Male infertility**   Onset = 20 |
| **Sex distribution** | In absence of information on the sex distribution of dioxin-induced hypothyroidy, a 50:50 age distribution was assumed.  For male infertility, the entire burden was assigned to males. |

**References**

1. Devleesschauwer B, Haagsma JA, Angulo FJ, Bellinger D, Cole D, Döpfer D, et al. (2015) Methodological framework for World Health Organization estimates of the global burden of foodborne disease. In preparation.
2. Vos T, Flaxman AD, Naghavi M, Lozano R, Michaud C, Ezzati M, et al. (2012) Years lived with disability (YLDs) for 1160 sequelae of 289 diseases and injuries 1990-2010: a systematic analysis for the Global Burden of Disease Study 2010. Lancet. 380(9859): 2163-2196.
3. Salomon JA, Haagsma JA, Davis A, Maertens de Noordhout C, Polinder S, Havelaar AH, et al. (2015) Disability weights for the Global Burden of Disease 2013 study. Lancet Glob Health. Submitted.
4. World Health Organization (2013) WHO methods and data sources for global burden of disease estimates 2000-2011. Global Health Estimates Technical Paper. WHO/HIS/HSI/GHE/2013.4. Available: <http://www.who.int/healthinfo/statistics/GlobalDALYmethods_2000_2011.pdf>.

# Peanut allergens

| **Incidence** | Data on clinically confirmed peanut allergy in children were available from six countries (i.e., Canada, UK, Denmark, Iceland, Sweden and Turkey). Average incidences ranged from 0 to 22.6 per 100,000 [1]. To reflect this uncertainty, the incidence rate of clinical peanut allergy in WHO A subregion countries was modelled as a Uniform distribution ranging from 0/100,000 to 22.6/100,000. Given the lack of data, no estimates were generated for other countries. |
| --- | --- |
| **Clinical outcomes** | The symptoms of peanut allergy vary from mild to severe, from swollen lips, shortness of breath to an anaphylactic shock, which is potentially fatal. However, because of the very short duration of acute peanut allergy, we decided not to include acute peanut allergy in the burden assessment. The considered clinical outcome was therefore living with peanut allergy and the anxiety of a possible allergic reaction. |
| **Duration** | It is assumed that peanut allergy is a lifelong disease. It is important to note that the duration of allergic symptoms is very short. |
| **Disability Weight** | Mullins et al. [2] reported that 52% of cases referred to a specialist allergy medical practice in Australia suffered from mild symptoms (skin and subcutaneous tissue involvement only), 42% from moderate symptoms (features suggestive of respiratory, cardiovascular or gastrointestinal involvement), and 6% from severe symptoms (cyanosis, hypotension, confusion, collapse, loss of consciousness, incontinence). We propose the DW for clinically relevant peanut allergy be a weighted average accounting for this severity distribution. GBD 2010 DWs [3] for the health states "Asthma: controlled" (DW=0.009) are considered applicable for mild and moderate cases (94%), and "Generic uncomplicated disease: anxiety about the diagnosis" (DW=0.054) for severe cases (6%), leading to a severity-weighted DW of 0.012 for clinically relevant peanut allergy. |
| **Mortality** | The limited data on the mortality rate of peanut-induced anaphylaxis show values ranging from 0 to 0.006 deaths per 100,000 person-years [1]. To reflect this uncertainty, the mortality rate of peanut-induced anaphylaxis in WHO A subregion countries was modelled as a Uniform distribution ranging from 0/100,000 to 0.006/100,000. Given the lack of data, no estimates were generated for other countries. |
| **Age distribution** | The onset of peanut allergy is early in life (median age 18-24 months, [4,5]), with continued prevalence in older age groups. All incident cases of peanut allergy were therefore assumed to develop early in life, i.e., before the age of five.  Deaths due to peanut allergen were assumed to occur at all ages, with an average age of 37 [1]. |
| **Sex distribution** | In absence of information on the sex distribution of peanut allergy, a 50:50 age distribution was assumed. |

**References**

1. Ezendam J, van Loveren H (2012) Parameters needed to estimate the global burden of peanut allergy: Systematic literature review. European Journal of Food Research & Review 2(2):46-48.
2. Mullins RJ, Dear KBG & Tang MLK (2009) Characteristics of childhood peanut allergy in the Australian Capital Territory, 1995 to 2007. Journal of Allergy and Clinical Immunology 123(3), 689-693.
3. Salomon JA, Vos T, Hogan DR, Gagnon M, Naghavi M, Mokdad A, et al. (2012) Common values in assessing health outcomes from disease and injury: disability weights measurement study for the Global Burden of Disease Study 2010. Lancet 380(9859): 2129-2143.
4. Sicherer SH, Furlong TJ, Munoz-Furlong A, Burks AW & Sampson HA (2001) A voluntary registry for peanut and tree nut allergy: characteristics of the first 5149 registrants. J Allergy Clin Immunol 108, 128-132.
5. Green TD, LaBelle VS, Steele PH, Kim EH, Lee LA, Mankad VS, Williams LW, Anstrom KJ & Burks AW (2007) Clinical Characteristics of Peanut-Allergic Children: Recent Changes. Pediatrics 120, 1304-1310.
