# Supplementary material for: World Health Organization estimates of the global and regional disease burden of four foodborne chemical toxins, 2010: a data synthesis
Source: F1000Res. 2015 Dec 3;4:1393. [Version 1] doi: 10.12688/f1000research.7340.1 (PMC4755404; doi:10.12688/f1000research.7340.1)
Supplement: Peer review history

# Peer review: Referee response for version 1

**DOI:** 10.5256/f1000research.7910.r12410

**Published:** 2016-02-12

**Authors:** Mary A Fox

**Affiliations:** 1 Department of Health Policy and Management, Johns Hopkins University Bloomberg School of Public Health, Baltimore, MD, USA

Gibb *et al*. have made an important contribution to our understanding of the population health impacts of food-borne chemical exposures. I noted one minor data gap in the description of the approach taken for the dioxin analysis.  In contrast to the other chemicals assessed, the authors did not report the disability weights (DWs) for dioxin outcomes in the main text; they are found only in the Supplementary material. 

                               

My main questions, however, relate to the conclusions. I feel that two of the paper’s bottom lines (on raising awareness of the impacts of food-borne chemicals and the need for better exposure data) deserve additional attention. 

On raising awareness: after reading the article, I was looking for some further characterization of the burden estimates. The estimates do seem substantial but what is the appropriate context for reference? The authors make reference to a Dutch National Institute for Public Health and the Environment assessment that made some comparisons of disease burdens for both chemicals and infectious agents in foods (listed as reference #12 Van Kreijl et al 2006). Perhaps that approach or some comparisons of the reported burden estimates to the total burden of the outcomes assessed could be made.  Some further characterization of the burden estimates would assist the effort to raise awareness in the public health community.

On better exposure data: The main text of the paper focuses largely on the outcomes or health effects related to the chemicals. Little is said about the exposure beyond an understanding that most exposures come from food.  Description of the types of exposure data represented in the literature underlying the analysis would better set up the call for biomonitoring at the conclusion.

I have read this submission. I believe that I have an appropriate level of expertise to confirm that it is of an acceptable scientific standard.

# Peer review: Referee response for version 1

**DOI:** 10.5256/f1000research.7910.r12086

**Published:** 2016-01-25

**Authors:** George M. Gray

**Affiliations:** 1 Department of Environmental and Occupational Health, George Washington University, Milken Institute School of Public Health, Washington, D.C, USA

This paper is a very useful addition to the goal of characterizing the disease burden from food contaminants.  It applies appropriate, and for some contaminants state-of-the-art, analytic approaches. The fact that virtually all exposure to the four contaminants evaluated comes from food illustrates the importance of the exercise. 

                                                              

There are a few issues that deserve attention:

- It is difficult to tell from the manuscript whether the dose-response information for dioxins is from the epidemiologic study cited or from the animal studies. It is unfortunate that further information on the dose response refers to a manuscript in preparation and thus unavailable.
- What is the appropriate weighting for a substance that causes infertility? Presumably some number of affected individuals would want to reproduce and the exposure is effectively causing an entire lifetime of YLL for the child not born. 
- I appreciate very much the effort to consider uncertainty in the projections from this analysis.  However, it is very important not to imply greater characterization of uncertainty than has occurred. In this analysis the uncertainty bounds presented are primarily based on ranges for specific parameters in the models used to estimate YLL and YLD. Model uncertainty, for example, is not considered.  Insofar as dose-response data for dioxins were generated from animal data (see point above) there is considerable quantitative uncertainty introduced by using animals as a model for humans. Similarly, in the case of aflatoxin it is recognized that a “bottom up” rather than “top down” model of analysis yields very different estimates of risk and uncertainty and it is not clear which is the better approach. Statements like that in paragraph 2 of the results section “The DALY estimates for aflatoxin and dioxin have the least uncertainty..” are likely to be misinterpreted. The smallest calculated uncertainty is not the same as the smallest range of actual uncertainty if all sources have not been considered. 

I have read this submission. I believe that I have an appropriate level of expertise to confirm that it is of an acceptable scientific standard.

# Peer review: Referee response for version 1

**DOI:** 10.5256/f1000research.7910.r11682

**Published:** 2016-01-05

**Authors:** Jonathan Spergel

**Affiliations:** 1 Division of Allergy, Children’s Hospital of Pennsylvania, Philadelphia, PA, USA

For the estimate of Konzo , it was multiple by 10.  I would suggest a range as it is an estimate based on poor reporting.  Is there another disease to model off to get a better range?

The rest of the article is acceptable.

I have read this submission. I believe that I have an appropriate level of expertise to confirm that it is of an acceptable scientific standard, however I have significant reservations, as outlined above.

# Response

**Published:** 2016-01-26

**Authors:** Herman Gibb

**Affiliations:** Gibb Epidemiology Consulting LLC, USA

In the section on cyanide in cassava, a range of 1 to 10-fold was reported:  "It was therefore decided to account for the uncertainty in the underreporting by applying an expansion factor ranging uniformly from 1 to 10 to the observed cases."
